# Evaluation of D-isomers of 4-borono-2-^18^F-fluoro-phenylalanine and *O*-^11^C-methyl-tyrosine as brain tumor imaging agents: a comparative PET study with their L-isomers in rat brain glioma

**DOI:** 10.1186/s13550-018-0404-6

**Published:** 2018-06-13

**Authors:** Masakatsu Kanazawa, Shingo Nishiyama, Fumio Hashimoto, Takeharu Kakiuchi, Hideo Tsukada

**Affiliations:** Central Research Laboratory, Hamamatsu Photonics K.K., 5000 Hirakuchi, Hamakita, Hamamatsu, Shizuoka 434-8601 Japan

**Keywords:** Boron neutron capture therapy, D-isomer, ^18^F-FBPA, ^11^C-CMT, Glioma

## Abstract

**Background:**

The potential of the D-isomerization of 4-borono-2-^18^F-fluoro-phenylalanine (^18^F-FBPA) to improve its target tumor to non-target normal brain tissue ratio (TBR) was evaluated in rat brain glioma and compared with those of L- and D-^11^C-methyl-tyrosine (^11^C-CMT).

The L- or D-isomer of ^18^F-FBPA was injected into rats through the tail vein, and their whole body kinetics and distributions were assessed using the tissue dissection method up to 90 min after the injection. The kinetics of L- and D-^18^F-FBPA or L- and D-^11^C-CMT in the C-6 glioma-inoculated rat brain were measured for 90 or 60 min, respectively, using high-resolution animal PET, and their TBRs were assessed.

**Results:**

Tissue dissection analyses showed that D-^18^F-FBPA uptake was significantly lower than that of L-^18^F-FBPA in the brain and abdominal organs, except for the kidney and bladder, reflecting the faster elimination rate of D-^18^F-FBPA than L-^18^F-FBPA from the blood to the urinary tract. PET imaging using ^18^F-FBPA revealed that although the brain uptake of D-^18^F-FBPA was significantly lower than that of L-^18^F-FBPA, the TBR of the D-isomer improved to 6.93 from 1.45 for the L-isomer. Similar results were obtained with PET imaging using ^11^C-CMT with a smaller improvement in TBR to 1.75 for D-^11^C-CMT from 1.33 for L-^11^C-CMT.

**Conclusions:**

The present results indicate that D-^18^F-FBPA is a better brain tumor imaging agent with higher TBR than its original L-isomer and previously reported tyrosine-based PET imaging agents. This improved TBR of D-^18^F-FBPA without any pre-treatments, such as tentative blood-brain barrier disruption using hyperosmotic agents or sonication, suggests that the D-isomerization of BPA results in the more selective accumulation of ^10^B in tumor cells that is more effective and less toxic than conventional L-BPA.

## Background

Boron neutron capture therapy (BNCT) is a biologically and physically targeted radiation therapy for tumors with the nuclear capture reaction of boron-10 (^10^B) specifically accumulating in tumor cells. The irradiation of low-energy thermal neutrons to ^10^B produces high-energy α (^4^He) particles and lithium ions (^7^Li), leading to cytotoxic effects against tumor cells. Due to the shorter track ranges of these particles (9 and 4 μm for α particles and ^7^Li ions, respectively) than the diameter of tumor cells, adjacent normal cells are expected to remain unaffected when these particles are emitted from tumor cells (for a review, see [1]). Two low molecular weight ^10^B carrier compounds, sodium sulfhydryl borane (BSH) [2] and L-4-boronophenylalanine conjugated with fructose (L-BPA-Fr) [3], have been clinically applied. BSH enters tumor cells by passive diffusion through the cell membrane [4–6]. L-BPA and its F-18-labeled PET probe L-^18^F-FBPA [7–12] are transported into tumor cells through L-type amino acid transporter 1 (LAT1) [11–13] and amino acid transporter B^0,+^ (ATB^0,+^) [13], both of which are strongly upregulated in tumor cells [13, 14]. Since L-BPA accumulates in tumor cells with a low target tumor to non-target normal brain tissue ratio (TBR) [6], L-^18^F-FBPA was developed to predict the exact concentration of ^10^B-BPA in tumor cells prior to BNCT [7–12]. The combined use of PET and inductively coupled plasma optical emission spectrometry confirmed that L-^18^F-FBPA-PET has the ability to estimate ^10^B concentrations delivered by L-BPA in the tumor and normal tissues of rats [10, 15] and humans [8].

In order to more effectively and safely perform BNCT, selective ^10^B delivery to tumor tissues with high TBR and sufficient thermal neutrons delivered to the tumor region are critical issues. One of the major limitations of L-BPA is its low TBR [6], which results in difficulties regulating the delivery of an adequate amount of ^10^B to target tumor tissues along with a sufficiently low amount of ^10^B to adjacent non-target normal tissues in order to avoid an adverse reaction. In an attempt to enhance L-BPA uptake into tumor cells, transient blood-brain barrier disruption (BBB-D) using hyperosmotic mannitol has been proposed to facilitate the uptake of L-BPA into F98 glioma in the rat brain, thereby enhancing survival times by BNCT over the non-BBB-D control [4, 15]. Another BBB-D by focused ultrasound sonication also increased the uptake of L-^18^F-FBPA into F98 glioma in the rat brain [16]. Since these BBB-D manipulations require treatments with drugs or sonication prior to the administration of L-BPA, the delivery of L-BPA into tumor cells may be affected by individual responses to these pre-treatments.

We previously demonstrated that the D-isomers of *O*-^11^C-methyl-tyrosine (^11^C-CMT), *O*-^18^F-fluoromethyl-tyrosine (^18^F-FMT), and *O*-^18^F-fluoroethyl-tyrosine (^18^F-FET) were better tumor imaging agents than their corresponding L-isomers in HeLa cells inoculated into the mouse hind leg [17–21]. In contrast to natural amino acid-based tumor imaging probes such as D-^11^C-methionine (D-^11^C-MET), the D-isomers of ^11^C-CMT, ^18^F-FMT, and ^18^F-FET were artificial amino acid-based probes without metabolic conversion from the D-isomer to L-isomer by D-amino acid oxidase [17, 22]. L-^11^C-MET converted from D-^11^C-MET via α-keto-γ-methiolbutyrate was incorporated not only into proteins via the conversion into amino-acyl-tRNA, but also into non-protein materials such as lipids and RNA by a transmethylation process via S-adenosyl-L-MET [23], leading to a worse TBR due to strong uptake into non-target normal tissues. The D-isomers of ^11^C-CMT, ^18^F-FMT, and ^18^F-FET exhibited higher TBRs than their corresponding L-isomers in HeLa cells inoculated into the mouse hind leg because of the faster elimination rate of D-isomers than L-isomers from the blood to the urinary tract [17, 18].

In the present study, we conducted the D-isomerization of ^18^F-FBPA in order to improve the properties of L-^18^F-FBPA as a brain tumor imaging agent with a higher TBR, and the kinetics and distributions of L- and D-^18^F-FBPA were compared in the whole body organs of normal F344 rats with a tissue dissection method as well as in C6 glioma inoculated into rat brains using PET. Furthermore, in order to establish whether the D-isomerization of amino acid-based PET probes may be generalized to improve TBR in the brain, the L- and D-isomers of ^11^C-CMT were also evaluated in rat brain C6 glioma.

## Methods

### Animals and chemicals

One hundred (60 for tissue dissection analyses; 32 for PET imaging; 8 for backup) male F344/NSlc rats (7 weeks of age; body weight of 126–149 g) were purchased from Japan SLC. Isoflurane and medetomidine hydrochloride were purchased from Dainippon Pharmaceutical and Nippon Zenyaku Kogyo, respectively. Midazolam and butorphanol tartrate were obtained from Sandoz and Meiji Seika Pharma, respectively. L-Tyrosine was obtained from Nacalai Tesque. AgOTf and D-tyrosine were from Sigma-Aldrich, Japan. L- and D-4-boronophenylalanine were purchased from Santa Cruz Biotechnology. Standard compounds of L- and D-FBPA were obtained from the NARD Institute. L- and D-isomers of CMT were purchased from Tokyo Chemical Industry.

### Implantation of tumor cells

Rats (8 weeks of age; body weight of 158–190 g) were anesthetized with a combination of three anesthetics (0.15 mg/kg of medetomidine hydrochloride, 2 mg/kg of midazolam, and 2.5 mg/kg of butorphanol tartrate) and placed in a stereotaxic apparatus. C6 glioma cells (2 × 10^5^ cells/5 μl of Ham’s F-12K) were injected at a rate of 0.5 μl/min into the right striatum of the rat (at the bregma, − 3.0 mm lateral to the midline suture, and − 5.0 mm from the cranium) via a microsyringe.

### PET ligand synthesis

Carbon-11 (^11^C) and fluorine-18 (^18^F) were produced by ^14^N(p, α)^11^C and ^20^Ne(d, α)^18^F nuclear reactions, respectively, using the cyclotron (HM-18, Sumitomo Heavy Industry). Labeled compounds were synthesized using a modified CUPID system (Sumitomo Heavy Industry). Chemical and radiochemical analyses of the labeled compounds were performed by HPLC consisting of a pump (LC20AT, Shimadzu), UV detector (SPD-20A, Shimadzu), radio detector (US-3000, Universal Giken), and a column (Inertsil ODS-3, GL-Science).

L- and D-^18^F-FBPA were produced as previously reported with minor modifications [6–12]. ^18^F-Acetylhypofluorite in Ne gas was bubbled at a flow rate of 50 mL/min at room temperature into 5 mL of trifluoroacetic acid containing 20 mg of L- or D-4-boronophenylalanine. Trifluoroacetic acid was removed under reduced pressure at 80 °C. The residue was also dissolved in 1.8 mL × 2 of water containing 0.1% acetic acid, and the solution was applied to HPLC (YMC-Pac ODS-A 20*150 mm 5 μm), with water for injection containing 0.1% acetic acid as the mobile phase. The fraction of L- or D-^18^F-FBPA was collected into an evaporation flask, evaporated to approximately 5 mL. The specific radioactivities of L- and D-^18^F-FBPA were 26.4 ± 3.7 and 28.3 ± 1.8 MBq/μmol, respectively, and the radiochemical purities of L- and D-^18^F-FBPA were more than 98%.

L- and D-^11^C-CMT were prepared by reactions of ^11^C-methyl iodide with the corresponding L- and D-tyrosine [17]. The specific radioactivities of L- and D-^11^C-CMT were 55.7 ± 14.1 and 58.6 ± 11.3 GBq/μmol, respectively, and their radiochemical purities were more than 98%.

Enantiomeric purity was analyzed on HPLC consisting of a pump (DP-8020, Tosoh), UV detector (UV-8020), radiodetector (TCS-913, Aloka), and column (CROWNPACK CR(+), Daicel) using perchloric acid (pH 2.0) as a mobile phase at a flow rate of 2 mL/min. Enantiomeric purities were more than 99% for the L- and D-isomers of ^11^C-CMT and ^18^F-FBPA.

### Tissue distribution assay

A total of 5 MBq of L- or D-^18^F-FBPA was injected into normal rats (*n* = 5/each time point) through the tail vein. Animals were killed by decapitation under isoflurane (1.5–2.0% in O_2_) anesthesia 1, 5, 10, 30, 60, and 90 min after the injection; samples of the brain, heart, lung, liver, kidney, spleen, pancreas, stomach, small intestine, colon, bladder, bone, muscle, and blood were rapidly removed, and their weights and radioactivities were measured using a γ-counter (1480 WIZARD, Perkin Elmer). Standardized uptake values (SUVs) were calculated as radioactivity in tissue divided by the ratio of total injected radioactivity and body weight.

In metabolite analyses, blood samples were centrifuged to separate plasma and then weighed, and radioactivity was measured; ethanol was added to plasma (sample:ethanol = 1:1) and centrifuged, and supernatants were developed on a thin-layer chromatography (TLC) plate (TLC Silica gel 60 F254, Merck) using a mobile phase of 1-propanol/30 mM ammonium acetate/acetic acid (4/2/0.01). The TLC plate was then transferred to a phosphoimaging plate (BAS-IIIs, Fuji film) for a 10-min exposure, and radioactivity was converted to digitalized imaging data using a Fuji BAS system (FLA-7000, Fuji film). The ratio of radioactivity in the unmetabolized fraction to that in total plasma (metabolized plus unmetabolized) was calculated.

### PET measurements

Fourteen days after the implantation of glioma cells, rats (*n* = 4–6/each condition) anesthetized by 1.5–2.0% isoflurane in O_2_ were positioned prone on a fixation plate and placed in the gantry hole of the PET scanner (SHR-38000, Hamamatsu Photonics). After a transmission scan for 15 min using a ^68^Ge-^68^Ga rotation rod source, the L- or D-isomer of ^18^F-FBPA or ^11^C-CMT at 10 MBq was intravenously injected into each rat via the tail vein, and this was followed by an emission scan for 90 min with ^18^F-FBPA or for 60 min with ^11^C-CMT. The body temperature of each animal was monitored throughout the study using a Thermo-Hygro Recorder (TR-72Ui, T&D Corporation).

PET data obtained were reconstructed using the dynamic row-action maximum likelihood algorithm method with a 1.0-mm Gaussian post filter [24], with attenuation corrections using transmission scan data. Dynamic images every 5 min for the time activity curve (TAC) as well as summation images from 60 to 90 min for ^18^F-FBPA and from 30 to 60 min for ^11^C-CMT were reconstructed, and SUV images were created as radioactivity in each pixel divided by the ratio of total injected radioactivity and body weight. Volumes of interest (VOIs) were set on the glioma region in the right brain hemisphere and the normal region in the contralateral left hemisphere, and TACs were obtained in each region as SUV. Improved magnitude of TBR by D-isomerization was determined as the ratio of TBR for D-isomer against TBR for corresponding L-isomer (TBR-D/TBR-L).

### Ex vivo autoradiography imaging of the brain

After PET imaging using L- or D-^18^F-FBPA, rat was sacrificed by cervical vertebral dislocation under anesthesia, and the brain was excised and sliced at a thickness of 2 mm using a brain slicer (Muromachi Kikai). Slices were transferred to a phosphoimaging plate (BAS-IIIs, Fuji film) for 10 min of exposure with an authentic standard radioactivity source. Radioactivity was converted to digitalized imaging data using a Fuji BAS system (FLA-7000, Fuji film).

### Histological assessment

Rats were sacrificed by cervical vertebral dislocation under anesthesia, and brains were excised. After fixation in 4% neutral-buffered formalin, samples were embedded in paraffin. Thirty-micrometer-thick brain slices were prepared with a cryotome (model CR-502; Nakagawa Seisakusyo), and slices were stained with hematoxylin and eosin (HE) for microscopic examinations.

### Statistical analysis

Results are expressed as means ± SD. Comparisons between conditions were performed using an unpaired, two-tailed Student’s *t* test. A probability level of less than 5% (*P* < 0.05) was considered to indicate significance.

## Results

**Fig. 1:**
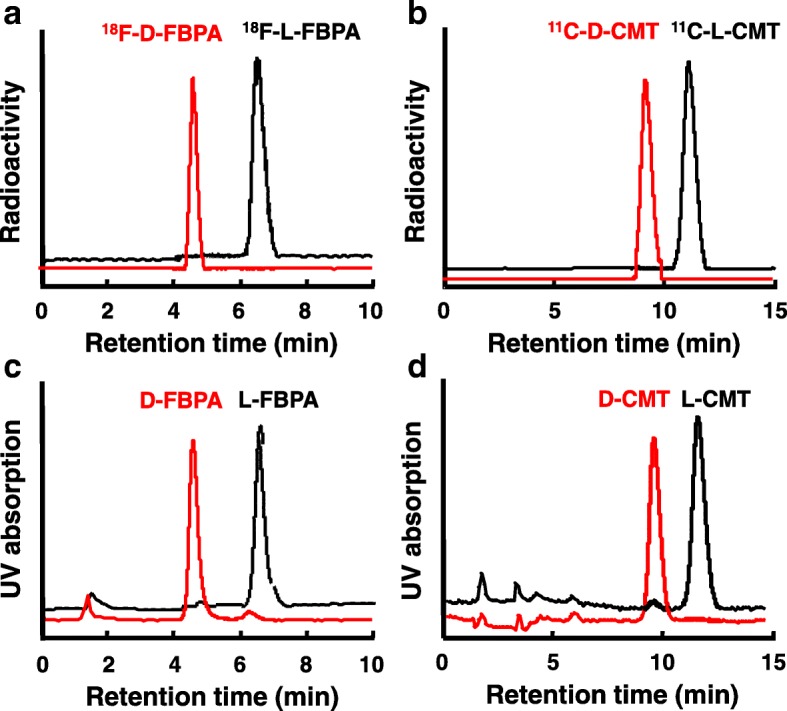
Analyses of enantiomeric purities of L- and D-isomers of ^18^F-FBPA (**c**) and ^11^C-CMT (**d**) used in PET measurements. The enantiomeric purity of each compound was analyzed on a CROWNPAK CR(+) column using an elution solution of perchloric acid (pH 2.0) at a flow rate of 2 mL/min. The identification of each compound was conducted by comparing the elution time to each corresponding authentic standard compound of L- and D-FBPA (**a**) and L- and D-CMT (**b**)

The analyzed data in Fig. 1 confirmed that ^18^F-FBPA and ^11^C-CMT preserved their enantiomeric specificities for the corresponding precursors after radiolabeling using F-18 or C-11. These retention times of 6.6 and 4.5 min for L- and D-^18^F-FBPA, respectively (Fig. 1c), and 11.1 and 9.2 min for L- and D-^11^C-CMT, respectively (Fig. 1d) were consistent with those obtained by the corresponding authentic standard compounds of L- and D-FBPA (Fig. 1a) and L- and D-CMT (Fig. 1b), respectively.

The kinetics of the L- and D isomers of ^18^F-FBPA in normal rats are summarized in Table 1. The blood level of D-^18^F-FBPA was significantly lower than that of L-^18^F-FBPA from 1 min after the injection throughout measurements. Metabolic analyses of plasma demonstrated that both isomers of ^18^F-FBPA were very stable, showing 90.0 ± 0.6 and 89.0 ± 2.4% of unmetabolized L- and D-^18^F-FBPA, respectively, 90 min after the injection. The SUVs of D-^18^F-FBPA in the heart, lung, liver, spleen, pancreas, stomach, small intestine, colon, bone, and muscle were lower than those of L-^18^F-FBPA throughout the study after the injection. The uptake of D-^18^F-FBPA into normal brain tissue was markedly low with 13% of L-^18^F-FBPA 90 min after the injection. The SUVs of D-^18^F-FBPA in the kidney and bladder were markedly higher than those of L-^18^F-FBPA throughout the study after the injection. Differences in whole body distributions between L- and D-^18^F-FBPA were generally similar to those between L- and D-^11^C-CMT (see Table 2 of [17]).

**Table 1:** Distribution and kinetics of L- and D-^18^F-FBPA in normal rats

	Organs	1 min	5 min	10 min	30 min	60 min	90 min
Brain	L	0.28 ± 0.20	0.29 ± 0.01	0.41 ± 0.02	0.60 ± 0.02	0.76 ± 0.05	0.77 ± 0.04
	D	0.15 ± 0.02	0.07 ± 0.01	0.05 ± 0.01	0.06 ± 0.01	0.07 ± 0.01	0.10 ± 0.01
Heart	L	1.23 ± 0.08	1.10 ± 0.03	1.15 ± 0.05	1.17 ± 0.05	1.10 ± 0.04	1.00 ± 0.04
	D	0.95 ± 0.08	0.81 ± 0.04	0.36 ± 0.03	0.24 ± 0.02	0.20 ± 0.02	0.21 ± 0.01
Lung	L	1.94 ± 0.25	1.56 ± 0.08	1.52 ± 0.15	1.20 ± 0.04	0.94 ± 0.02	0.92 ± 0.05
	D	1.92 ± 0.15	1.04 ± 0.07	0.75 ± 0.04	0.48 ± 0.03	0.34 ± 0.03	0.32 ± 0.01
Liver	L	1.69 ± 0.06	2.01 ± 0.05	2.16 ± 0.05	1.68 ± 0.07	1.40 ± 0.04	1.27 ± 0.06
	D	1.16 ± 0.07	1.42 ± 0.04	1.64 ± 0.09	1.67 ± 0.04	1.19 ± 0.08	0.80 ± 0.04
Kidney	L	10.56 ± 0.65	11.57 ± 0.69	10.88 ± 0.97	9.57 ± 0.47	9.11 ± 0.77	8.15 ± 0.62
	D	18.37 ± 1.13	37.61 ± 1.66	47.82 ± 1.17	38.30 ± 0.81	25.56 ± 3.32	19.97 ± 1.76
Spleen	L	1.83 ± 0.07	2.08 ± 0.08	2.16 ± 0.21	1.77 ± 0.13	1.46 ± 0.04	1.33 ± 0.05
	D	0.88 ± 0.06	0.66 ± 0.04	0.44 ± 0.02	0.44 ± 0.01	0.43 ± 0.04	0.44 ± 0.02
Pancreas	L	2.67 ± 0.35	2.80 ± 0.88	3.05 ± 0.29	2.40 ± 0.22	2.00 ± 0.24	1.75 ± 0.21
	D	1.08 ± 0.15	0.94 ± 0.14	1.24 ± 0.13	1.15 ± 0.08	0.80 ± 0.12	0.73 ± 0.04
Stomach	L	1.26 ± 0.16	1.62 ± 1.05	1.14 ± 0.08	1.12 ± 0.08	0.94 ± 0.04	0.86 ± 0.05
	D	1.06 ± 0.07	0.81 ± 0.05	0.53 ± 0.06	0.35 ± 0.02	0.28 ± 0.04	0.28 ± 0.02
S. intestine	L	1.64 ± 0.09	1.51 ± 0.07	1.58 ± 0.04	1.33 ± 0.08	1.20 ± 0.06	1.08 ± 0.07
	D	0.90 ± 0.02	0.51 ± 0.02	0.50 ± 0.04	0.69 ± 0.06	0.78 ± 0.03	0.73 ± 0.07
Colon	L	0.73 ± 0.19	0.51 ± 0.04	0.61 ± 0.08	0.62 ± 0.08	0.57 ± 0.01	0.54 ± 0.02
	D	0.48 ± 0.04	0.33 ± 0.03	0.26 ± 0.03	0.16 ± 0.01	0.15 ± 0.02	0.15 ± 0.03
Bladder	L	0.85 ± 0.19	1.29 ± 0.12	1.17 ± 0.22	1.60 ± 0.46	1.75 ± 0.33	1.84 ± 0.33
	D	0.98 ± 0.26	1.46 ± 0.24	1.92 ± 0.63	5.16 ± 2.13	4.79 ± 2.37	4.46 ± 1.82
Bone	L	1.00 ± 0.09	1.05 ± 0.05	1.09 ± 0.04	1.04 ± 0.09	0.97 ± 0.01	0.94 ± 0.04
	D	1.01 ± 0.09	1.01 ± 0.05	0.71 ± 0.03	0.70 ± 0.03	0.61 ± 0.02	0.57 ± 0.01
Muscle	L	0.33 ± 0.02	0.36 ± 0.02	0.48 ± 0.01	0.65 ± 0.05	0.75 ± 0.02	0.78 ± 0.04
	D	0.28 ± 0.06	0.27 ± 0.03	0.22 ± 0.01	0.12 ± 0.01	0.09 ± 0.01	0.11 ± 0.01
Blood	L	2.96 ± 0.04	2.09 ± 0.04	1.86 ± 0.02	1.03 ± 0.04	1.10 ± 0.02	0.99 ± 0.05
	D	0.55 ± 0.06	1.15 ± 0.07	0.84 ± 0.05	0.56 ± 0.02	0.42 ± 0.03	0.38 ± 0.01

Rats (*n* = 5/each time point) were injected intravenously with 5 MBq of L- or D-^18^F-FBPA via the tail vein and killed at each time point after the injection; tissue samples were rapidly removed, and their weights and radioactivities were measured. Data are expressed as mean ± SD of SUV

**Fig. 2:**
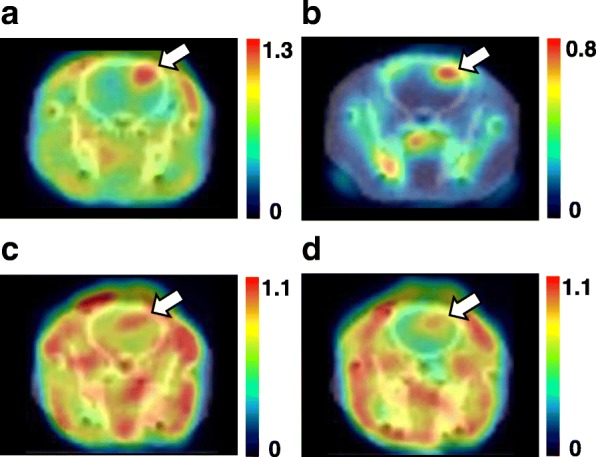
PET imaging using L- and D-^18^F-FBPA (**a**, **b**) and L- and D-^11^C-CMT (**c**, **d**) in C6 glioma-bearing rat brains. Rats anesthetized by isoflurane were positioned prone on a fixation plate. After a transmission scan for 15 min, 10 MBq of each PET probe was intravenously injected, and an emission scan was performed for 90 min with ^18^F-FBPA or for 60 min with ^11^C-CMT. Summation images from 60 to 90 min for ^18^F-FBPA and from 30 to 60 min for ^11^C-CMT were reconstructed, and SUV images were created

As shown in Fig. 2, PET imaging of rat C6 glioma indicated that the L- and D-isomers of ^18^F-FBPA (a and b) and ^11^C-CMT (c and d) facilitated the detection of the tumor region in the rat brain. Although glioma cells were injected in the striatum as shown in the “Methods” section, the tumor tissues in almost all rat brains were observed in the cortex as shown in Fig. 2. It is assumed that the injected cells might draw back from the striatum to the cortex through the track of injection needle due to the high pressure in the brain, and then, the cells that were retained in the cortex proliferated.

The absolute uptakes, expressed as SUV, of the D-isomers of ^18^F-FBPA (Fig. 2b) and ^11^C-CMT (Fig. 2d) were lower in the tumor and non-target normal tissue regions of the brain than those of the corresponding L-isomers (Fig. 2a, c); however, the image contrast reflecting TBR appeared to be markedly better for D-isomers (Fig. 2b, d) than for the corresponding L-isomers (Fig. 2a, c).

**Fig. 3:**
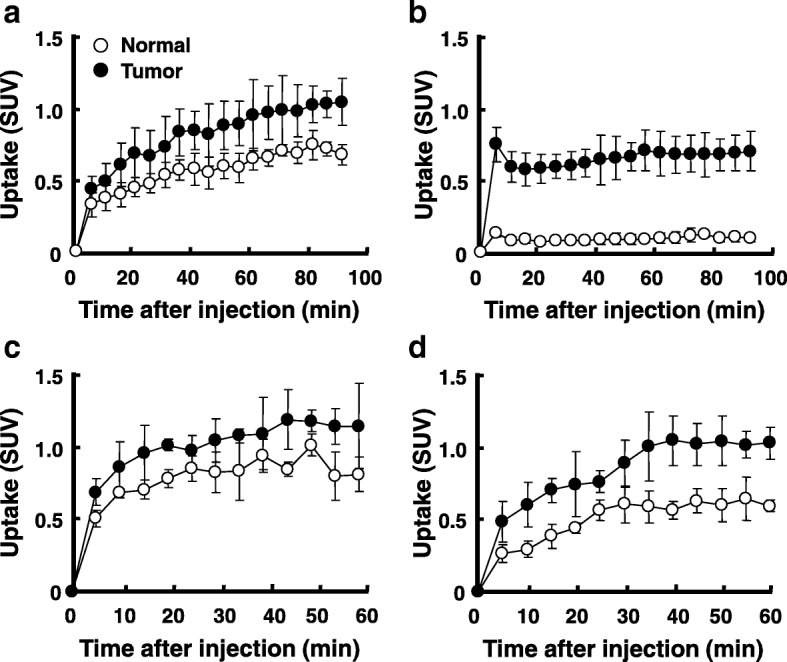
Time activity curves (TACs) of L- and D-^18^F-FBPA (**a**, **b**) and L- and D-^11^C-CMT (**c**, **d**) in C6 glioma-bearing rat brains. PET scans were performed as shown in the legend of Fig. 2, dynamic images were reconstructed every 5 min for TACs, VOIs were set on the glioma region in the right brain hemisphere and normal region in the contralateral left hemisphere, and TACs were obtained in each region as SUVs

As shown in Fig. 3a, L-^18^F-FBPA exhibited slow and gradual uptake over time in tumor and normal regions up to 90 min after the injection. In contrast, although the uptake levels of D-^18^F-FBPA were low, TAC remained constant in tumor and normal tissues from 10 min and up to 90 min after the injection (Fig. 3b). The TACs of L- and D-^11^C-CMT gradually increased and then remained constant in tumor and normal tissues 40 min and more after the injection (Fig. 3c, d).

**Fig. 4:**
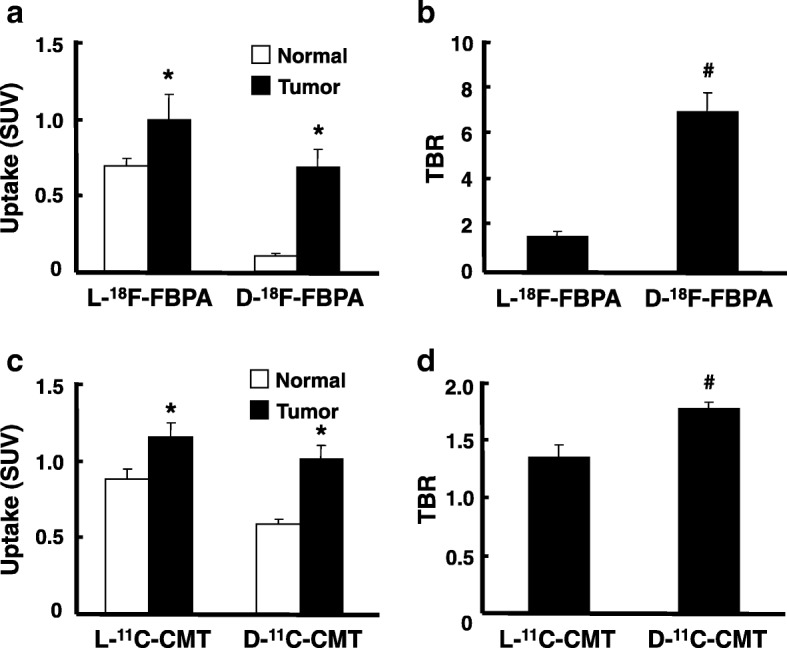
Uptakes (**a**, **c**) and TBRs (**b**, **d**) of L- and D-^18^F-FBPA (**a**, **b**) and L- and D-^11^C-CMT (**c**, **d**) into C6 glioma-bearing rat brains. PET scans were performed as shown in the legend of Fig. 2, and the VOIs of glioma and contralateral normal regions were set on the summation SUV images from 60 to 90 min for ^18^F-FBPA (**a**) and from 30 to 60 min for ^11^C-CMT (**c**). The target tumor (glioma) to non-target (normal) ratios of L- and D-^18^F-FBPA (**b**) and L- and D-^11^C-CMT (**d**) were calculated. *P < 0.05 vs. vehicle. ^**#**^*P* < 0.05 vs. L-isomer

The uptakes of the D-isomers of ^18^F-FBPA and ^11^C-CMT into brain glioma were 68 and 89% of the corresponding L-isomers, while the uptakes of D-isomers into non-target normal brain tissues were only 15 and 68% of the corresponding L-isomers (Fig. 4a, c). The important results of the present study are shown in Fig. 4b, d, namely, TBRs were improved by D-isomerization from 1.45 to 6.93 for ^18^F-FBPA (TBR-D/TBR-L = 4.76) (b) and from 1.33 to 1.75 for ^11^C-CMT (TBR-D/TBR-L = 1.38) (d).

**Fig. 5:**
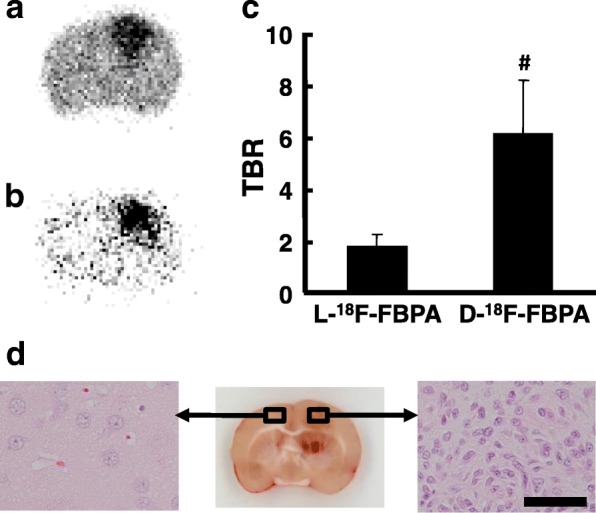
Ex vivo autoradiography and histochemical imaging of C6 glioma-bearing rat brains. After PET imaging using L- (**a**) or D-^18^F-FBPA (**b**), rats were sacrificed under anesthesia, the brain was excised and sliced at a thickness of 2 mm, and the slices were then exposed to a phosphoimaging plate in order to calculate the target tumor (glioma) to non-target (normal) ratios of L- and D-^18^F-FBPA (**c**). ^**#**^*P* < 0.05 vs. L-isomer. The brain excised from another rat bearing C6 glioma was fixed in 4% neutral-buffered formalin; samples were embedded in paraffin, sliced at a thickness of 30 μm, and stained with HE (**d**)

The rat brains were dissected into slices for an autoradiographic examination just after the PET imaging. As shown in Fig. 5, both L- (a) and D-^18^F-FBPA (b) accumulated less in the normal region of the brain, and thus, the tumor regions corresponding to HE-positive area (c) were clearly imaged. As expected from the PET imaging data, although the uptake of D-^18^F-FBPA into glioma was lower than that of L-^18^F-FBPA, D-isomer uptake into non-target normal tissue was markedly lower than that of the L-isomer, resulting in TBR values of 6.12 and 1.74 for D-^18^F-FBPA and L-^18^F-FBPA, respectively (Fig. 5d). These results were consistent with those obtained in PET imaging analyses shown in Fig. 4.

## Discussion

The present study demonstrated that the D-isomers of ^18^F-FBPA and ^11^C-CMT had higher TBRs in rat brain C6 glioma than the corresponding L-isomers. In tumor imaging using amino acid-based PET probes, their transport through upregulated LAT1 [13, 19, 25, 26] and ATB^0,+^ [13] is important for their high uptake into tumor cells, and the uptake of D-^18^F-FBPA into tumor cells may depend on the activity of these transporters, as reported previously for L-^18^F-FBPA [11, 12] and L- and D-^11^C-CMT [19]. Our previous study using mice inoculated with HeLa cells into their hind legs demonstrated that the uptakes of D-^11^C-CMT into the normal brain region and abdominal organs, except for the kidney and bladder, were significantly lower than those of L-^11^C-CMT [17], which was consistent with the present results obtained using L- and D-^18^F-FBPA. In contrast to the similar uptake levels of L- and D-^11^C-CMT into HeLa cells inoculated into the mouse hind leg [17], the uptakes of D-^11^C-CMT and D-^18^F-FBPA into glioma cells inoculated into the rat brain were lower than those of the corresponding L-isomers. There were several differences between the two assay conditions: the species used (mouse vs. rat), tumor cells (HeLa vs. C6 glioma), and inoculated regions (hind leg vs. brain). Among these differences, we confirmed that HeLa and C6 glioma cells both strongly expressed LAT1 [19]. In addition to the species difference, the involvement of BBB needs to be taken into account for the different kinetic properties between the L- and D-isomers. The effects of the D-isomerization of amino acid-based PET probes on BBB penetration appear to be controversial; one study showed no selective transport between L- and D-^11^C-MET in normal and glioma regions [27], whereas another indicated the lower uptake of D-^11^C-MET than L-^11^C-MET in normal and astrocytoma regions [28]. Furthermore, the K1 (rate constant of amino acid transport) of D,L-^18^F-FBPA in normal and glioma regions was lower than that of L-^18^F-FBPA [8]. It is important to note that these three studies found no significant differences in TBRs between the L- and D (D,L) isomers [8, 27, 28]. The present results demonstrated that the uptake levels of D-^18^F-FBPA and D-^11^C-CMT into normal brain regions were 15 and 68%, respectively, of their corresponding L-isomers and also that the uptakes of these D-isomers into brain C6 glioma were 69 and 89%, respectively, of the corresponding L-isomers. These different uptake profiles between the L- and D-isomers in normal and tissue regions resulted in improved TBRs from 1.45 (L-^18^F-FBPA) to 6.93 (D-^18^F-FBPA) (TBR-D/TBR-L = 4.76) and from 1.33 (L-^11^C-CMT) to 1.75 (D-^11^C-CMT) (TBR-D/TBR-L = 1.38). The faster washout of D-isomers than L-isomers from blood resulted in limited tumor uptake due to shorter available time for its uptake into tumor, while the lower background activity was the advantage for higher TBRs. The TBR of D-^18^F-FBPA (6.93) was markedly higher than that of L-^18^F-FET ranging from 2.0 (U87 glioblastoma) [29] to 3.1 (F98 glioma) [9] in rat brain glioma.

A critical point for more beneficial BNCT is to achieve a high delivery amount of ^10^B to tumor cells; however, it is considered to be more important to maximize the TBR of ^10^B uptake for proper therapeutic efficacy in tumors with less damage to normal tissues. L-BPA is currently used as a ^10^B carrier molecule to tumors, and although some preliminary evidence for the therapeutic effectiveness of BNCT using L-BPA has been reported, clinical outcomes are still considered to be unsatisfactory [30, 31]. The major limitation of BNCT efficacy is the relatively low TBR of ^10^B uptake [6]. Tentative BBB-D using hyperosmotic agents or sonication has been proposed to facilitate the brain tumor uptake of L-BPA/^18^F-FBPA [4, 15]. When an intracarotid injection of L-BPA was combined with a pretreatment with hyperosmotic mannitol, the TBR of L-BPA was 1.3–1.6-fold higher in F98 glioma than in the non-treated control [4, 15]. However, in addition to BBB-D, the administration of mannitol increases the glomerular filtration rate and renal blood flow [32], which may affect the kinetics and delivery of L-BPA to tissues due to a change in the excretion rate from the blood to the urinary tract. Another pretreatment with focused ultrasound sonication also enhanced the TBR of ^18^F-FBPA into the brain tumor by up to 1.75-fold that of the non-treated control [16]. One of the difficulties associated with BBB-D is possible neurotoxicity that may result in the exposure of normal brain tissues to toxic materials. Although L-BPA is not known to be neurotoxic, BBB-D further disseminates ^10^B-BPA as well as ^18^F-FBPA not only to tumor cells, but also to normal brain tissues that have an intact BBB before manipulations. Furthermore, since these BBB-D manipulations require pre-treatments with drugs or sonication prior to the administration of BPA, the delivery of BPA into tumor cells may be affected by individual responses to these pre-treatments, which are difficult to predict prior to BNCT. In contrast, the present results suggested that D-isomerization improved the TBRs of ^18^F-FBPA in C6 glioma without any pre-treatments. Based on the higher TRB of D-^18^F-FBPA than L-^18^F-FBPA, the D-isomerization of BPA may have potential as a method to improve the TBR of ^10^B uptake. Further animal studies are needed not only to investigate the effects of the D-isomerization of BPA on TBR improvement, but also to evaluate treatment efficacy with BNCT.

## Conclusions

The present study demonstrated the potential of D-^18^F-FBPA as a better brain tumor imaging agent with higher TBR than its original L-isomer and previously reported tyrosine-based PET imaging agents. D-isomerization was more effective for ^18^F-FBPA than ^11^C-CMT to improve TBR for brain tumor imaging agents. This improved TBR of D-^18^F-FBPA without any pre-treatments, such as tentative BBB-D using hyperosmotic agents or sonication, suggests that the D-isomerization of BPA results in the more selective accumulation of ^10^B in tumor cells that is more effective and less toxic than conventional L-BPA.

## References

[1] Pisarev MA, Dagrosa MA, Juvenal GJ (2007). Boron neutron capture therapy in cancer: past, present and future. Arq Bras Endocrinol Metabol.

[2] Soloway AH, Hatanaka H, Davis MA (1967). Penetration of brain and brain tumor. VII. Tumor-binding sulfhydryl boron compounds. J Med Chem.

[3] Mishima Y, Honda T, Ichihashi M, Obara H, Hiratsuka J, Fukuda H, Karashima H, Kobayashi T, Kanda K, Yoshino K (1989). Treatment of malignant melanoma by single thermal neutron capture therapy with melanoma-seeking ^10^B-compound. Lancet.

[4] Barth RF, Yang W, Bartus RT, Moeschberger ML, Joel DD, Nawrocky MM, Goodman JH, Soloway AH (1997). Boron neutron capture therapy of brain tumors: enhanced survival following intracarotid injection of either sodium borocaptate or boronophenylalanine with or without blood-brain barrier disruption. Cancer Res.

[5] Yoshida F, Matsumura A, Yamamoto T, Kumada H, Nakai K (2004). Enhancement of sodium borocaptate (BSH) uptake by tumor cells induced by glutathione depletion and its radiobiological effect. Cancer Lett.

[6] Yokoyama K, Miyatake S, Kajimoto Y, Kawabata S, Doi A, Yoshida T, Asano T, Kirihata M, Ono K, Kuroiwa T (2006). Pharmacokinetic study of BSH and BPA in simultaneous use for BNCT. J Neuro-Oncol.

[7] Ishiwata K, Ido T, Mejia AA, Ichihashi M, Mishima Y (1991). Synthesis and radiation dosimetry of 4-borono-2-[^18^F]fluoro-D,L-phenylalanine: a target compound for PET and boron neutron capture therapy. Int J Rad Appl Instrum A.

[8] Imahori Y, Ueda S, Ohmori Y, Kusuki T, Ono K, Fujii R, Ido T (1998). Fluorine-18-labeled fluoroboronophenylalanine PET in patients with glioma. J Nucl Med.

[9] Wang H-E, Wu S-Y, Chang C-W, Liu R-S, Hwang L-C, Lee T-W, Chen J-C, Hwang J-J (2005). Evaluation of F-18-labeled amino acid derivatives and [^18^F]FDG as PET probes in a brain tumor-bearing animal model. Nucl Med Biol.

[10] Hanaoka K, Watabe T, Naka S, Kanai Y, Ikeda H, Horitsugi G, Kato H, Isohashi K, Shimosegawa E, Hatazawa J (2014). FBPA PET in boron neutron capture therapy for cancer: prediction of ^10^B concentration in the tumor and normal tissue in a rat xenograft model. EJNMMI Res.

[11] Watabe T, Ikeda H, Nagamori S, Wiriyasermkul P, Tanaka Y, Naka S, Kanai Y, Hagiwara K, Aoki M, Shimosegawa E, Hatazawa J (2017). ^18^F-FBPA as a tumor-specific probe of L-type amino acid transporter 1 (LAT1): a comparison study with ^18^F-FDG and ^11^C-methionine PET. Eur J Nucl Med Mol Imaging.

[12] Yoshimoto M, Kurihara H, Honda N, Kawai K, Ohe K, Fujii H, Itami J, Yasuaki Arai Y (2013). Predominant contribution of L-type amino acid transporter to 4-borono-2-^18^F-fluoro-phenylalanine uptake in human glioblastoma cells. Nucl Med Biol.

[13] Wongthai P, Hagiwara K, Miyoshi Y, Wiriyasermkul P, Wei L, Ohgaki R, Kato I, Hamase K, Nagamori S, Kanai Y (2015). Boronophenylalanine, a boron delivery agent for boron neutron capture therapy, is transported by ATB^0,+^, LAT1 and LAT2. Cancer Sci.

[14] Kanai Y, Endou H (2001). Heterodimeric amino acid transporters: molecular biology and pathological and pharmacological relevance. Curr Drug Metab.

[15] Hsieh C-H, Chen Y-F, Chen F-D, Hwang J-J, Chen J-C, Liu R-S, Kai J-J, Chang C-W, Wang H-E (2005). Evaluation of pharmacokinetics of 4-borono-2-^18^F-fluoro-L-phenylalanine for boron neutron capture therapy in a glioma-bearing rat model with hyperosmolar blood-brain barrier disruption. J Nucl Med.

[16] Yang F-Y, Chang W-Y, Li J-J, Wang H-E, Chen J-C, Chang C-W (2014). Pharmacokinetic analysis and uptake of ^18^F-FBPA-Fr after ultrasound-induced blood-brain barrier disruption for potential enhancement of boron delivery for neutron capture therapy. J Nucl Med.

[17] Tsukada H, Sato K, Fukumoto D, Nishiyama S, Harada N, Kakiuchi T (2006). Evaluation of D-isomers of *O*-^11^C-methyl tyrosine and *O*-^18^F-fluoromethyl tyrosine as tumor-imaging agents in tumor-bearing mice: comparison with L- and D-^11^C-methionine. J Nucl Med.

[18] Tsukada H, Sato K, Fukumoto D, Kakiuchi T (2006). Evaluation of D-isomers of *O*-^18^F-fluoromethyl, *O*-^18^F-fluoroethyl and *O*-^18^F-fluoropropyl tyrosine as tumour imaging agents in mice. Eur J Nucl Med Mol Imaging.

[19] Urakami T, Sakai K, Asai T, Fukumoto D, Tsukada H, Oku N (2009). Evaluation of *O*-[^18^F]fluoromethyl-D-tyrosine as a radiotracer for tumor imaging with positron emission tomography. Nucl Med Biol.

[20] Murayama C, Harada N, Kakiuchi T, Fukumoto D, Kamijo A, Kawaguchi AT, Tsukada H (2009). Evaluation of D-^18^F-FMT, ^18^F-FDG, L-^11^C-MET, and ^18^F-FLT for monitoring the response of tumors to radiotherapy in mice. J Nucl Med.

[21] Zitzmann-Kolbe S, Strube A, Frisk A-L, Kakonen S-M, Tsukada H, Hauff P, Berndorff D, Graham K (2010). D-^18^F-Fluoromethyl tyrosine imaging of bone metastases in a mouse model. J Nucl Med.

[22] Stegink LD, Moss J, Printen KJ, Cho ES (1980). D-methionine utilization in adult monkeys fed diets containing DL-methionine. J Nutr.

[23] Ishiwata K, Vaalburg W, Elsinga PH, Paans AMJ, Woldring MG (1988). Comparison of L-[1-^11^C]methionine and L-methyl-[^11^C]methionine for measuring in vivo protein synthesis rates with PET. J Nucl Med.

[24] Tanaka E, Kudo H (2010). Optimal relaxation parameters of DRAMA (dynamic RAMLA) aiming at one-pass image reconstruction for 3D-PET. Phys Med Biol.

[25] Vaalburg W, Coenen HH, Crouzel C, Elsinga PH, Långström B, Lemaire C, Meyer GJ (1992). Amino acids for the measurement of protein synthesis in vivo by PET. Nucl Med Biol.

[26] Ishiwata K, Kawamura K, Wang WF, Furumoto S, Kubota K, Pascali C, Bogni A, Iwata R (2004). Evaluation of O-[^11^C]methyl-L-tyrosine and O-[^18^F]fluoromethyl-L-tyrosine as tumor imaging tracers by PET. Nucl Med Biol.

[27] Schober O, Duden C, Meyer GJ, Muller JA, Hundeshagen H (1987). Non selective transport of [^11^C-methyl]-L- and D-methionine into a malignant glioma. Eur J Nucl Med.

[28] Bergström M, Lundqvist H, Ericson K, Lilia A, Johnström P, Långström B, von Holst H, Eriksson L, Bromqvist G (1987). Comparison of the accumulation kinetics of L-(methyl-^11^C)-methionine and D-(methyl-^11^C)-methionine in brain tumor studies with positron emission tomography. Acta Radiol.

[29] Stegmayr C, Oliveira D, Niemietz N, Willuweit A, Lohmann P, Galldiks N, Shah NJ, Ermert J, Langen K-J (2017). Influence of bevacizumab on blood-brain barrier permeability and O-(2-^18^F-fluoroethyl)-L-tyrosine uptake in rat gliomas. J Nucl Med.

[30] Nakagawa Y, Hatanaka H (1997). Boron neutron capture therapy: clinical brain tumor studies. J Neuro-Oncol.

[31] Joensuu H, Kankaanranta L, Seppala T, Auterinen I, Kallio M, Kulvik M (2003). Boron neutron capture therapy of brain tumors: clinical trials at the Finnish facility using boronophenylalanine. J Neuro-Oncol.

[32] Behnia R, Koushanpour E, Brunner EA (1996). Effects of hyperosmotic mannitol infusion on hemodynamics of dog kidney. Anesth Analg.

